# Effects of helminth co-infections on atopy, asthma and cytokine production in children living in a poor urban area in Latin America

**DOI:** 10.1186/1756-0500-7-817

**Published:** 2014-11-19

**Authors:** Neuza Maria Alcântara-Neves, Gabriela de S G Britto, Rafael Valente Veiga, Camila A Figueiredo, Rosimeire Leovigildo Fiaccone, Jackson S da Conceição, Álvaro Augusto Cruz, Laura Cunha Rodrigues, Philip John Cooper, Lain C Pontes-de-Carvalho, Maurício Lima Barreto

**Affiliations:** Departamento de Ciências da Biointeração, Instituto de Ciências da Saúde, Universidade Federal da Bahia, Salvador, Brazil; Instituto de Matemática, Universidade Federal da Bahia, Salvador, Brazil; Instituto de Saúde Coletiva, Universidade Federal da Bahia, Salvador, Brazil; ProAR – Nucleo de Excelência em Asma, Universidade Federal da Bahia, Salvador, Brazil; London School of Hygiene and Tropical Medicine, University of London, London, UK; Escuela de Biologia, Pontifícia Universidad Católica Del Ecuador, Quito, Ecuador; Institute of Infection and Immunity, St George’s University of London, London, UK; Centro de Pesquisas Gonçalo Moniz, Fundação Oswaldo Cruz, Salvador, Brazil; Instituto de Ciências da Saúde, Universidade Federal da Bahia, Avenida Reitor Miguel Calmon, sem n°, Canela, CEP – 40110-100 Salvador, Bahia Brazil

**Keywords:** Helminth infections, Eosinophilia, Total IgE, Atopy, Asthma, Cytokines, Treg cells

## Abstract

**Background:**

Helminths are modulators of the host immune system, and infections with these parasites have been associated with protection against allergies and autoimmune diseases. The human host is often infected with multiple helminth parasites and most studies to date have investigated the effects of helminths in the context of infections with single parasite or types of parasites (e.g. geohelminths). In this study, we investigated how co-infections with three nematodes affect markers of allergic inflammation and asthma in children. We selected *Ascaris lumbricoides and Trichuris trichiura*, two parasites that inhabit the human intestine and *Toxocara* spp (*Toxocara canis* and/or *T. cati*), intestinal roundworms of dogs and cats that cause systemic larval infection in humans. These parasites were selected as the most prevalent helminth parasites in our study population.

**Results:**

36.4% of children were infected with one parasite; 12.7% with 2 and 5.2% with 3. Eosinophilia >4% and >10% was present in 74.3% and 25.5% of the children, respectively. Total IgE > 200 IU/mL, sIgE ≥ 0.70 kU/L and SPT positivity were present in 59.7%, 37.1% and 30% of the children, respectively. 22.7% had recent asthma (12.0% non-atopic and 10.7% atopic). Helminth infections were associated in a dose-dependent way to decrease in the prevalence of SPT and increase in eosinophilia, total IgE, and the production of the regulatory cytokine IL-10 by unstimulated peripheral blood leukocytes. No association with asthma was observed.

**Conclusions:**

Helminth co-infections in this population were associated with increased markers of the Th2 immune response, and with a host immune regulatory phenotype that may suppress allergic effector responses such as immediate hypersensitivity reactions in the skin.

## Background

Infections with helminths that inhabit the vasculature or tissues of the host such as *Schistosoma* spp. and filarial parasites have potent modulatory effects on the immune system of humans and experimental animals [1]. In children, such infections have been associated with the suppression of the immune response to vaccines [2], decreased skin hypersensitivity to aeroallergens [3, 4], a milder form of asthma [5], and a reduction of inflammation in an animal model of autoimmune disease [6].

Although some studies in humans have shown that infections with intestinal helminths such as *Ascaris lumbricoides* and *Trichuris trichiura* are associated with immune modulation [7] and the downregulation of atopy [8], studies of the effect of these parasites on asthma prevalence have been inconsistent, with some studies demonstrating a reduced prevalence [9], others no association [10], and others an increased risk [11, 12]. These discrepancies have been attributed to differences between populations with respect to the parasites present, timing of first infections, size of worm burdens and infection chronicity [1]. For example, Rodrigues and collaborators [8] showed that children who had infections with *T. trichiura* in early childhood had a reduced prevalence of skin test reactivity to aeroallergens later in childhood, while in the same population, Alcântara-Neves and collaborators [11] reported a positive association between *T. trichiura* infection and wheeze symptoms when the children were of pre-school age.

The prevalence of infection with intestinal helminths is decreasing in large cities of developing countries, where sanitation has been introduced [13]. However, infections with *Toxocara* spp. (*T. canis* and *T. cati*) are highly prevalent in such environments, since they are not affected by sanitation. A decrease in the prevalence of toxocariasis depends on the control of stray animals and antihelminthic treatment of pets to prevent the exposure of children to this parasite, neither of which are easy to achieve [14]. In the Brazilian city of Salvador, where the present study was done, Datolli and collaborators [15] found a *Toxocara* infection seroprevalence of 46% in blood donors who were not infected with intestinal helminths. Seropositive individuals were more likely to have elevated allergic markers of blood eosinophilia and total IgE. Previous studies have indicated that individuals with toxocariasis may have an increased risk of atopy and asthma [16].

We have shown previously that pathogens causing chronic infections, including intestinal helminths [17] and *Toxocara* spp [18], can modulate atopy in children, but not wheezing. We have also shown that *Ascaris lumbricoides* and *Trichuris trichiura* in this population are positively associated to IL-10 production by non-stimulated whole blood cells [7]. In the present study, we investigated the effects of single and co-infections with intestinal parasites (*A. lumbricoides* and *T. trichiura*) and *Toxocara* spp. on the following outcomes: blood eosinophils total and allergen-specific IgE, skin reactivity to aeroallergen, atopic and non-atopic asthma and cytokine responses, in children living in poor neighborhoods of a Brazilian city.

## Results

### Frequencies of study variables

Of the 1,445 children enrolled in this study, 1,271 with complete outcome data were analyzed. Analyses for associations with eosinophilia were done for 1,155 of the latter with data for this variable. No statistically significant differences were seen between the 174 excluded children and those included with respect to important baseline variables (data not shown). The analyzed children were aged between 4 and 11 years with the following age distribution: ≤5 years (25.9%), between 6–7 (40.5%) and >7 (33.5%). 54% of the children were male; 70.2% of mothers had not completed second grade; and parental asthma was reported for 13.5% of children. With respect to helminth infections: 15.8% were infected with *A. lumbricoides*, 13.8% with *T. trichiura* and 47.8% were seropositive for *Toxocara* spp IgG antibodies; 45.6% of children had no helminth infection, 36.4% had one, 12.7% had two and 5.2% had infections with three different helminth parasites. The prevalence of allergic-type outcomes in the study population were: eosinophilia of >4% and >10% was observed in 74.3% and 25.5% of children, respectively; total IgE ≥200 IU/mL was present in 59.7%; sIgE ≥ 0.70 kU/L and SPT positivity for at least one allergen were found in 37.1% and 30% respectively; 22.7% had asthma, with 12% having non-atopic asthma, and 10.7% atopic asthma; 26% of children were atopic but not asthmatic (data not shown).

### Association of helminth infections with markers of immediate hypersensitivity and asthma

The number of helminth infections was positively and statistically associated with eosinophilia at >4 and >10% and the presence of elevated total IgE, in a dose-dependent manner (Table 1). Table 2 shows that there was no statistically significant association between helminth infections and the presence of sIgE but there was a statistically significant inverse association with SPT positivity, which was stronger with increasing number of infections. Table 3 shows that the number of helminth infections was not significantly associated with asthma, irrespective of atopic status, although a non-significant trend of increased risk was observed for non-atopic asthma.

**Table 1:** **Associations between number of different helminth parasite infections and eosinophilia or total IgE in 1,155 children**

Number of helminth infections (n = 1,155)	Eosinophilia > 4%		Eosinophilia > 10%		Total IgE >200 IU/mL	
	n (%)	#OR	n (%)	#OR	n (%)	#OR
		(95% CI)		(95% CI)		(95% CI)
0	343 (64.6)		83 (15.6)		282 (52.9)	
(n = 533; 46.2%)		1		1		1
1	336 (79.6)	**2.13**	110 (26.1)	**1.81**	268 (63.5)	**1.53**
(n = 422; 36.6%)		**(1.57; 2.90)**		**(1.31; 2.52)**		**(1.17; 1.99)**
2	121 (87.1)	**3.36**	62 (44.6)	**3.82**	96 (69.1)	**1.92**
(n = 139; 12.0%)		**(1.96; 5.77)**		**(2.51; 5.83)**		**(1.28; 2.88)**
3	58 (95.1)	**8.85**	39 (63.9)	**7.96**	44 (72.1)	**2.22**
(n = 61; 5.3%)		**(2.70; 28.99)**		**(4.42; 14.35)**		**(1.22; 4.02)**

#Adjusted for gender, age, maternal education and parental asthma. Numbers in bold are statistically significant at P < 0.05. OR – odds ratio. 95% CI – 95% confidence interval.

**Table 2:** **Associations between number of different helminth parasite infections and specific IgE or skin prick test (SPT) positivity for at least one allergen in 1,271 children**

Number of helminth infections	^#^ sIgE ≥0.70		^#^ SPT	
	n (%)	*OR	n (%)	*OR
		(95% CI)		(95% CI)
0				
(n = 580; 45.6%)	212 (36.6)	1	202 (34.8)	1
1		1.09		**0.71**
(n = 463; 36.4%)	175 (37.8)	(0.84; 1.41)	128 (27.7)	**(0.54; 0.94)**
2		1.15		**0.60**
(n = 162; 12.8%)	64 (39.6)	(0.79; 1.67)	40 (24.7)	**(0.40; 0.91)**
3		0.79		**0.36**
(n = 66; 5.2%)	21 (31.8)	(0.45; 1.38)	11 (16.7)	**(0.18; 0.71)**

^#^For at least one the four studied allergens; *Adjusted for gender, age, maternal education and parental. Numbers in bold are statistically significant at P < 0.05. OR – odds ratio. 95% CI – 95% confidence interval.

**Table 3:** **Associations between number of different helminth parasite infections and asthma or asthma phenotypes in 1,271 children**

Number of helminth infections	All asthma		Non-atopic asthma		Atopic asthma		
	n (%)	*OR (95% CI)	n (%)	**Non-atopic/non-asthmatic	n (%)	**Non-atopic/non-asthmatic	**Atopic/non-asthmatic
0							
(n = 580; 45.6%)	164 (28.3)	1	59 (10.2)	1	60 (10.3)	1	1
1		1.02		1.40		1.24	1.01
(n = 463; 36.4%)	133 (28.7)	(0.78; 1.34)	60 (13.0)	(0.92; 2.11)	51 (11.0)	(0.81; 1.90)	(0.69; 1.74)
2		1.20		1.33		1.23	1.01
(n = 162; 12.8%)	52 (32.1)	(0.82; 1.75)	21 (13.0)	(0.74; 2.38)	19 (11.7)	(0.68; 2.23)	(0.53; 1.92)
3		1.55		1.84		0.91	1.02
(n = 66; 5.2%)	25 (37.9)	(0.91; 2.63)	12 (18.2)	(0.87; 3.89)	6 (9.1)	(0.36; 2.34)	(0.37; 2.84)

*Adjusted for gender, age, maternal education and parental asthma; ** Reference groups for polytomous logistic regression analysis. OR – odds ratio. 95% CI – 95% confidence interval.

### Association of helminth infection with cytokine production by peripheral blood leukocytes

**Figure 1:**
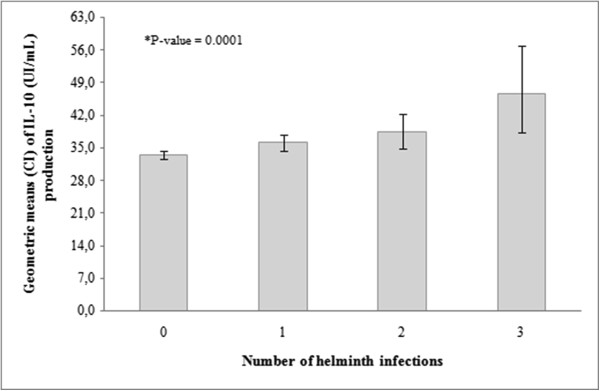
**Geometric means (pg/mL) and standard deviation (SD) of IL-10 production by non-stimulated blood cells in the IL-10 responder children, by number of of helminth infections.** Kruskal-Wallis test (P = 0.0001).

Tables 4 and 5 show associations between having one or more helminth infections and cytokine production by peripheral blood leukocytes (PBLs) in whole blood cultures stimulated with *A. lumbricoides* (Table 4) or in the presence of medium alone (i.e. unstimulated) (Table 5). In whole blood cultures stimulated with *A. lumbricoides* antigen, the production of IL-5 was positively associated with increasing number of helminth infections. Although IL-10 was associated with helminth infections, this association was not significant after adjustment for confounders; and IL-13 production was significantly associated only with having three helminth infections. In the case of spontaneous cytokine production, IL-10, but none of the other cytokines, was associated with helminth infections, i.e., children with increasing number of infections were more likely to be IL-10 cytokine responders. Figure 1 shows that the geometric mean IL-10 production in unstimulated PBL cultures increased with increasing number of helminth parasite infections (P = 0.0001).

**Table 4:** **Associations between number of different helminth parasite infections and cytokine production by peripheral blood leukocytes (PBLs) stimulated with** ***A. lumbricoides antigen*** **in 1,271 children**

Number of helminth infections	***A. lumbricoides*** -induced cytokine production				
	n (%)	% Non-responders	% Responders	Crude	Adjusted
		(n)	(n)	^#^ OR (95% CI)	^##^ OR (95% CI)
**IFN-γ** [mean (pg/mL) = 12.3]*					
		(n = 904)	(n = 23)		
0	416 (44.9)	97.1	2.9	1	1
1	342 (36.9)	98.0	2.0	0.70 (0.27; 1.81)	0.71 (0.27; 1.87)
2	114 (12.3)	97.4	2.6	0.91 (0.25; 3.28)	0.90 (0.24; 3.38)
3	55 (5.9)	98.2	1.8	0.62 (0.08; 4.89)	0.64 (0.08; 5.23)
**IL-5** [mean (pg/mL) = 26.6]*					
		(n = 1.013)	(n = 139)		
0	530 (46,0)	91.3	8.7	1	1
1	422 (36,6)	87.7	12.3	1.47 (0.97; 2.25)	1.45 (0.94; 2.23)
2	140 (12,2)	82.9	17.1	**2.17 (1.28; 3.71)**	**1.89 (1.09; 3.28)**
3	60 (5,2)	71.7	28.3	**4.16 (2.20; 7.87)**	**3.54 (1.82; 6.86)**
**IL-10** [mean (pg/mL) = 36.3]*					
		(n = 1.205)	(n = 51)		
0	568 (45.2)	97.4	2.6	1	1
1	461 (36.7)	95.1	4.9	**1.93 (1.00; 3.75)**	1.87 (0.95; 3.66)
2	160 (12.8)	93.8	6.2	**2.46 (1.08; 5.58)**	2.23 (0.96; 5.18)
3	67 (5.3)	95.5	4.5	1.73 (0.49; 6.13)	1.43 (0.39; 5.23)
**IL-13** [mean (pg/mL) = 64.5]*					
		(n = 955)	(n = 236)		
0	542 (45.5)	81.6	18.4	1	1
1	438 (36.8)	79.2	20.8	1.16 (0.84; 1.59)	1.18 (0.85; 1.63)
2	147 (12.3)	83.0	17.0	0.91 (0.56; 1.47)	0.90 (0.55; 1.48)
3	64 (5.4)	68.8	31.2	**2.01 (1.13; 3.56)**	**1.99 (1.10; 3.58)**

^#^Crude logist analysis. ^##^Logist analysis adjusted for gender, age, maternal education and parental asthma. *Geometric mean; Numbers in bold are statistically significant at P < 0.05 . OR – odds ratio. 95% CI – 95% confidence interval.

**Table 5:** **Associations between number of different helminth parasite infections and cytokine production by peripheral blood leukocytes cultured in medium alone (i.e. spontaneous cytokine production) in 1,271 children**

Number of helminth infections	Spontaneous cytokine production				
	n (%)	% Non-responders	% Responders	Crude	Adjusted
		(n)	(n)	^#^ OR (95% CI)	^##^ OR (95% CI)
**IFN-γ** [mean (pg/mL) = 21.0]*					
		(n = 870)	(n = 114)		
0	450 (445.7)	89.8	10.2	1	1
1	361 (36.7)	87.8	12.2	1.22 (0.79; 1.89)	1.17 (0.74; 1.84)
2	117 (11.9)	86.3	13.7	1.39 (0.76; 2.56)	1.35 (0.72; 2.53)
3	56 (5.7)	85.7	14.3	1.46 (0.65; 3.28)	1.36 (0.59; 3.11)
**IL-5** [mean (pg/mL) = 16.0]*					
		(n = 1.150)	(n = 66)		
0	569 (46.8)	95.1	4.9	1	1
1	443 (36.4)	94.1	5.9	1.20 (0.70; 2.09)	1.18 (0.67; 2.07)
2	144 (11.8)	95.8	4.2	0.84 (0.34; 2.07)	0.86 (0.34; 2.15)
3	60 (5.0)	90.0	10.0	2.15 (0.85; 5.41)	2.18 (0.84; 5.67)
**IL-10** [mean (pg/mL) = 35.6]*					
		(n = 1.213)	(n = 110)		
0	610 (46.1)	95.1	4.9	1	1
1	482 (36.4)	90.7	9.3	**1.99 (1.23; 3.21)**	**1.99 (1.22; 3.24)**
2	164 (12.4)	88.4	11.6	**2.53 (1.39; 4.63)**	**2.45 (1.32; 4.55)**
3	67 (5.1)	76.1	23.9	**6.07 (3.10; 11.86)**	**5.97 (2.97; 12.01)**
**IL-13** [mean (pg/mL) = 85.6]*					
		(n = 826)	(n = 432)		
0	542 (45.5)	65.4	34.6	1	1
1	458 (36.4)	66.6	33.4	0.95 (0.73; 1.23)	0.96 (0.73; 1.24)
2	152 (12.1)	64.5	35.5	1.04 (0.72; 1.51)	1.07 (0.73; 1.57)
3	64 (5.0)	64.1	35.9	1.06 (0.62; 1.82)	1.10 (0.63; 1.91)

^#^Crude logist analysis. ^##^Logist analysis adjusted for gender, age, maternal education and parental asthma. *Geometric mean; Numbers in bold are statistically significant at P < 0.05. OR – odds ratio. 95% CI – 95% confidence interval.

## Discussion

This study was conducted in Salvador, a city located in the northeastern coastal region of Brazil with a population of over 2.8 million and with a high reported prevalence of asthma in previous surveys [19]. To study the potential role of helminth infections in the high prevalence of allergy and asthma, we explored the effects of single or multiple helminth co-infections with *A. lumbricoides*, *T. trichiura* and *Toxocara* spp., on the occurrence of several allergy-associated outcomes. We observed a positive association between number of helminth infections and peripheral blood eosinophilia, elevated total IgE, spontaneous production of IL-10 and helminth antigen-stimulated production of Th2 cytokines. The number of helminth infections was inversely associated with allergen SPT and positively associated with IL-10 production by non-stimulated blood cells, but had no significant effect on the risk of asthma.

Studies of the associations between helminth infections and allergy have shown inconsistent findings. In general, an increased risk of asthma has been associated with *A. lumbricoides* infection [20] while reduced allergen SPT has been associated with *T. trichiura*[8] and hookworm infections [20, 21], although some studies were unable to demonstrate an association [22]. Systemic helminth infections (e.g. *Schistosoma* spp) have been associated with a decreased prevalence of SPT [3, 4, 22] a mild form of asthma (5) and protection against airways hiperreactivity in experimental models [6]. However, *Toxocara* spp, which are not natural infections of humans in whom they are unable to develop beyond the larval stage, but do cause systemic infections in the form of visceral larva migrans, have been associated with increased atopy [23] and asthma [24] in some, but not all studies [25]. We have reported previously, in the same population of children as the present study that: 1) human infections with *Toxocara*[18] and intestinal helminths [8, 17] were inversely associated with SPT; 2), *T. trichiura* infection and the presence of anti-*Ascaris* IgE were associated with atopic asthma in the early childhood; and 3) heavy infections with *T. trichiura* in early childhood were associated with protection against SPT in later childhood [8].

Although helminth parasite infections may protect their hosts against inflammatory diseases, they have also been associated with significant morbidity and economic losses through effects on nutrition, impaired school performance [26] and reduced efficacy of routine vaccinations [2]. A better understanding of parasite effects on the host immune response is, therefore, of considerable relevance not only to improve strategies for helminth control but also for the development of novel treatments for inflammatory diseases such as allergic and autoimmune diseases based on the isolation of parasite molecules with therapeutic potential [6, 27]. Previous studies of the effects of infections on atopy and allergy have studied the role of viral, bacterial and protozoal infections in developed countries [28–30] and one study from a developing country investigated viral and bacterial infections in the context of endemic helminth infections [17]. However, to the best our knowledge, no previous study has examined the effects of co-infections with intestinal and systemic helminth infections on the modulation of atopic and allergic disease markers, as done in the present study.

Helminth infection are believed to induce an immune response which is distinct from that produced in response to other chronic pathogens (e.g. protozoa, viruses and bacteria) in that chronic helminth infections and co-infections [1, 7, 31, 32] are characterized by a Th2 immune response in which various Th2 inflammatory markers such as eosinophilia and increased serum IgE levels are prominent. In this study, we observed increased production of Th2 cytokines by PBLs stimulated with parasite antigen from helminth-infected children, which was more evident with increasing number of helminth parasite infections. These data thus support the large body of literature showing that helminth infections induce Th2-type immune responses [31]. The prominent IgE response againt helminth infection, that is predominantly polyclonal, has been suggested to play a role in the down-modulation of skin reactivity to allergens through the saturation of high-affinity IgE receptors (FcϵRI) on mast cells, thus limiting the potential for interaction between allergens and specific IgE on mast cells [3, 33]. However, it has been shown that interference of allergen-specific response by polyclonal IgE only occurs at very high levels of IgE that are considered not to be commonly present in human populations [34]. We have tested this hypothesis by analyzing the correlation between levels of total IgE with skin test reactivity determined by wheal size; however, as the total IgE positive children of our study presented in general medium to low levels of total IgE we did not found a negative association between these outcomes.

In the present study, we observed a robust association between helminth co-infections and frequency or levels of spontaneous (or unstimulated) IL-10 production by PBLs. Meanwhile, although the helminth-antigen induced Th2 cytokine (mostly IL-5) responses by these cells were high, we have found relatively few IL-10 responses. The effect on spontaneous IL-10 production was greater with increasing number of helminth infections. We hypothesize that spontaneous IL-10 may play a role in suppressing effector function of mast cells and, consequently, skin prick test reactivity.

**Figure 2:**
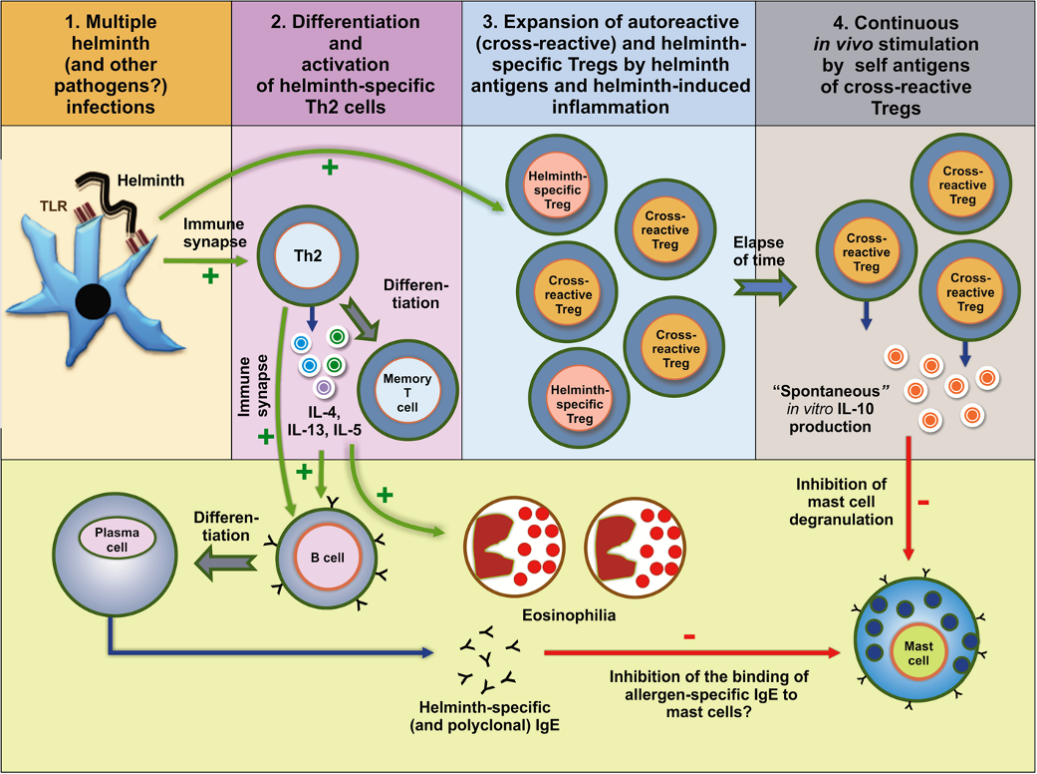
**A hypothesis to explain the pattern of cytokine production observed in cultures of peripheral blood leukocytes from individuals who had been infected with multiple helminths, specifically spontaneous production of IL-10 in the absence of exogenous antigen (i.e. in unstimulated cultures); and the presence of a Th2 recall response, but not an IL-10 recall response, to** ***A. lumbricoides*** **antigens. (1)** Multiple helminth infections could lead to stimulation of the immune system by hundreds of different antigens, including antigens that are cross-reactive with self antigens. **(2)** Strong helminth-specific and nonspecific polyclonal Th2 immune responses, with the differentiation and expansion of memory cells, would result from such infections. The Th2 immune response could lead to helminth-specific and non-specific polyclonal IgE production, to eosinophil expansion and activation, and to inflammation. The high levels of polyclonal IgE could theoretically inhibit mast cell activation, by competition with allergen-specific IgE for FcϵRI receptors on mast cells, causing inhibition of immediate hypersensitivity skin test to allergens). **(3)** A consequent differentiation and expansion of helminth-specific Tregs, some of them cross-reactive with self antigens (and some perhaps only autoantigen-reactive, as a consequence of tissue damage by inflammation) could occur (for the sake of clarity, only cross-reactive and helminth-specific Tregs are shown). **(4)** The cross-reactive/self antigen-specific Tregs would be maintained in a steady state of activation by self antigens, and would release low levels of regulatory cytokines, including IL-10. The spontaneous production of elevated levels of IL-10 could suppress mast-cell degranulation. Because these Tregs could be maintained through continuous stimulation to self antigens *in vivo*, they would not be further stimulated by helminth antigens *in vitro*. TLR- toll-like receptor.

Infections with complex organisms such as helminths and other parasites are likely to stimulate the immune system with a large number of antigens. The greater the number of infections the greater the overall stimulation and such stimulation could expand and activate populations of regulatory T cells (Tregs) [35]. One hypothesis, proposed by our research group, is that many such Tregs may recognize helminth antigens that share cross-reactivity with autoantigens, which in turn could maintain the Tregs in an activated state (Figure 2). Infection with helminths have been associated with increased numbers of circulating CD4^+^ CD25^+^ FoxP3^+^ Tregs and of autoantigen-stimulated PBLs that produce IL-10 and TGF-β, observations that have been associated with an improved clinical course in patients with multiple sclerosis [36]. Further, improvements in hygiene, associated with fewer infections with parasites and other pathogens, have been associated with a greater prevalence of autoimmune diseases [37]. The increased stimulation of cross-reactive Tregs due to past or present helminth infections could explain the spontaneous production of IL-10 observed in the present study. Because these cells may have been stimulated *in vivo,* they may suffer apoptosis when stimulated by *A. lumbricoides* antigens *in vitro.* This might explain our observation for the failure of *A. lumbricoides* antigen to induce IL-10 production by PBLs from helminth-infected children *in vitro*. This hypothesis has been reviewed in detail elsewhere [38]. In fact, we have observed previously, in this study population, an increased frequency of the production of IL-10 spontaneously among the children living in conditions of poor hygiene and with intestinal helminth infections [7, 39]. The induction of regulatory mechanisms, exemplified by elevated production of the immune regulatory cytokine IL-10 by unstimulated cells, has been attributed to living in environments with low hygiene and intense microbial and helminth exposure during childhood – the decline in such exposures in many populations living in affluent countries and the increase in the prevalence of inflammatory diseases is considered to be a consequence of a failure in microbe/parasite-induced immune regulation [40].

As expected, no association was found between helminth co-infections and IFN-γ production by PBLs spontaneously and in response to *A. lumbricoides* antigen. In addition, no statistically significant association was observed for sIgE, although a trend of decreasing frequency of sIgE risk was observed with increasing number of infections. It appears, therefore, that helminth infections do not affect sIgE but do up-regulate helminth-specific or non-specific polyclonal IgE. However, we cannot rule out that some helminth could be up regulating while others could be downregulating sIgE. As an exemple, *Toxocara* spp was associated with increased sIgE in a previous study reported by our group [18, 34].

Although atopic markers are modulated by helminth infection, no association was found between helminth co-infections and asthma (atopic and non-atopic). Asthma is a complex disease and differences in asthma phenotypes between different populations may explain differences in the effects of helminth infections on asthma observed between studies [41]. The lack of association between asthma and parasites found in this study may be result of the predominance of non-atopic asthma found in this population [42], since many studies have shown that infections, including those caused by helminths, protect against atopy and atopic asthma [1, 3, 4, 17].

## Conclusion

In summary, our data show strong and positive associations between helminth infections, eosinophilia, and total IgE and an inverse association between helminth infections and SPT in helminth-infected children. To our knowledge, this is the first study to show that an increasing number of helminth infections induce a dose–response effect on these allergic inflammatory markers. Our data demonstrate also that helminth infection, and especially multiple infections, are associated with a Th2 immune response (e.g. the production of Th2 cytokines by PBLs stimulated with helminth antigens and peripheral blood eosinophilia) but that such infections are associated with the upregulation of a regulatory network with an increase in unstimulated IL-10 production which could play a role in the suppression of immediate hypersensitivity reactions in the skin. Further studies are needed to understand the molecular mechanisms by which this immune modulation occurs and they could test our hypothesis that helminth infections in early childhood may induce or upregulate populations of regulatory immune cells that recognize cross-reactive self antigens and serve to dampen inflammatory responses to both endogenous and exogenous stimuli.

## Methods

### Study design, population and data collection

We analyzed data collected from 1,271 out of 1,445 children aged 4–11 years, enrolled in the SCAALA Program (Social Change in Asthma and Allergies in Latin America) [43]. One hundred and seventy-four of the 1,445 children were excluded because of missing data. The study population comprised a cohort of children living in 24 micro-areas in poor neighborhoods scattered throughout the city, originally constituted to evaluate the impact of a sanitation program on health outcomes when the children were aged 0–4 years. Cohorts of different children were recruited in 1999, 2000 and 2002, with a total of 2,973 children [44]. In these surveys, the child’s guardians were interviewed using a standard questionnaire to collect data on housing, sanitation, and socioeconomic conditions [43]. Allergic outcomes were evaluated in the cohort when the children were aged 4–11 years in 2005 using an ISAAC phase II questionnaire translated into Portuguese.

For this study, we evaluated a sample of 1,445 children of the original 2,973 children from the 24 poorest micro-areas. A questionnaire was administered during a home visit in 2005 to collect data from guardians on asthma in the child and, social, demographic and environmental data. Ethical approval was provided by the Ethics Committee of the Instituto de Saúde Coletiva, Universidade Federal da Bahia and by the Brazilian National Ethics Committee. Written, informed consent detailing all procedures to be carried out on the children was signed by a parent or the legal guardian of each child.

### Blood collection and skin prick test (SPT)

The children were evaluated by a medical team composed of a doctor, a nurse and a laboratory technician, in a mobile clinic, that visited each study neighborhood, where blood was collected and skin prick testing for relevant aeroallergens was done. Heparinized blood was collected for differential blood cell counts using in an automated counter (Counter Electronics, Hialeah, FL, USA), for the preparation of plasma used for measurement of total and allergen-specific IgE (sIgE) and IgG antibodies against specific pathogens, including *Toxocara* spp, and for whole blood cultures for measurement of cytokines.

SPTs were done on the right forearm of each child using extracts of *Dermatophagoides pteronyssinus*, *Blomia tropicalis*, *Blattella germanica*, *Periplaneta americana*, fungi, cat and dog danders (all from ALK-Abello, São Paulo, Brazil). Saline and 10 mg/mL histamine solution were used as negative and positive controls, respectively. Reactions were read after 15 minutes and a mean wheal diameter size of at least 3 mm greater than the negative control was considered positive. Frequencies of positive skin test reactions to dog and cat epithelia and a fungal allergen mix were low (<4%) and were excluded from further analysis.

### Definition of asthma and atopy

Children were classified as having asthma if parents/guardians reported wheezing in the previous 12 months and at least one of the following: diagnosis of asthma ever, wheezing with exercise in the last 12 months, four or more episodes of wheezing in the last 12 months, and waking up at night because of wheezing in the last 12 months. These criteria are more specific than wheezing in the last 12 months, the most commonly used definition in the ISAAC studies. All other children were classified as non-asthmatic.

Since the prevalence of sIgE for each of the studied allergens was greater than the SPT prevalence and the frequencies of SPT positivity among those without sIgE was very low [fungi (0.5%), dog epithelium (1.1%) and cat epithelium (0.9%)], atopy was defined as the presence of at least one sIgE ≥0.70 kU/L irrespective of SPT results. Atopic and non-atopic asthma cases were defined by the presence of symptoms of asthma as defined above in the presence or absence, respectively, of sIgE ≥0.70 kU/L for any of the tested aeroallergens.

#### Laboratory measurements

**Parasitological analysis** Two stool samples were collected from each child two days apart and analyzed using the gravitational sedimentation and Kato-Katz [45] techniques to detect eggs of *A. lumbricoides, T. trichiura*, hookworms and *S. mansoni*. Because hookworm (0.1%), *Enterobius vermiculares* (1.4%), *Strongyloides stercoralis* (0%) and *S. mansoni* (0%), were rare or absent in the study population, they were excluded from the analysis and only *A. lumbricoides* and *T. trichiura* were considered in the present analysis.

**Detection of total and specific IgE** The measurement of total IgE was performed as previously described [7]. The assay cut-off (200 IU/mL) was the 75^th^ centile of values obtained from 54 sera from children with three negative stool samples collected serially, specific IgE levels <0.35 kU/L, and <0.2% eosinophilia in peripheral blood.

Measurement of specific IgE (sIgE) against *B. tropicalis, D. pteronyssinus, P. americana, B. germanica* was done using the ImmunoCAP assay (Phadia Diagnostics AB, Uppsala, Sweden). These mite and cockroach allergens were chosen to measure atopy based on previous findings from allergen skin prick testing that showed these to be the most frequently recognized aeroallergens in our study population. A serum sample was considered positive if sIgE for any of the four allergens was ≥0.70 kU/L.

**Absorption of sera with*A. lumbricoides*and*T. trichiura*extracts and detection of serum anti-*Toxocara*IgG antibodies** To eliminate reactivity against antigenic determinants shared by the ascarid worms *A. lumbricoides* and *Toxocara* spp., human sera were absorbed with somatic antigen of *A. lumbricoides* before measurement of anti-*Toxocara* IgG. The *A. lumbricoides* somatic antigen was prepared as described previously [15]. Because 10.7% of the children were infected with *T. trichiura*, some samples of the studied sera were also absorbed with this parasite extract and compared to the same sera absorbed with *A. lumbricoides* alone or with both parasites, but because absorption with *A. lumbricoides* alone or with both parasites provided comparable titers of anti-*Toxocara* IgG, the remaining sera were absorbed with *A. lumbricoides* antigen alone.

The detection of anti-*Toxocara* IgG antibodies was carried out as previously described by Savigny and Tizard [46], with modifications, as reported previously [15]. Excretory/secretory products of *Toxocara canis* larvae (TcESLA) used in this assays were obtained as described previously [47]. The cut-off of this assay was the mean plus three standard deviations of results obtained from sera from 20 negative control children with no history of contact with dogs and cats, attending a private hospital. Because this assay does not discriminate infection caused by *T. canis* or *T. cati,* we used the results of this assay as marker of past or present infection with both or any of the two *Toxocara* species [48]. Although serum positivity does not necessarily imply current infection, we have used in this study the presence of IgG antibodies for *Toxocara* spp to indicate parasite infection.

**Cytokine production by whole blood cultures stimulated with medium alone or with*A. lumbricoides*** Whole blood cultures were done as described previously [7]. Briefly, we collected venous blood into heparinized tubes and cultured the collected cells at a dilution of 1:4 in RPMI (Gibco, Auckland, NZ) containing 10 mM glutamine (Sigma-Aldrich, Inc., Saint. Louis, Missouri, USA) and 100 μg/ml gentamicin (Sigma-Aldrich, Inc., Saint. Louis, Missouri, USA). The cells were cultured within 6 hours of collection and were maintained in a humidified environment of 5% CO_2_ at 37°C for 24 hours for detection of IL-10 and 5 days for the other cytokines in the presence or absence of *Ascaris lumbricoides* antigen (10 ug/mL). Cytokine concentrations were measured using commercial assays as recommended (R & D Systems, Minneapolis, USA). The detection limits (low/high) in pg/mL for each cytokine were as follows: IL-5 (15.6/500), IL-13 (62.5/4,000), IFN-γ (18.5/300), and IL-10 (31.3/500). The concentration values of the cytokine production by the antigen-stimulated cells were obtained after subtraction by the concentration values of the respective cytokine production by non-stimulated cells (background). Responders were defined as those children with cytokine concentrations above the lower detection limits.

### Statistical analysis

For associations between burden of helminthic infection and eosinophilia, total IgE, aeroallergen-specific IgE and SPT, and asthma and cytokine responder status, univariate and multivariate analyses were done using logistic regression with robust estimators for micro-areas to adjust for clustering. However, because models with and without adjustment for clustering showed similar results only unadjusted results are shown. Comparisons levels of IL-10 by unstimulated cells in whole blood cultures by number of helminth parasite infections was done using the Kruskal-Wallis test.

For the associations between helminth coinfection and the different asthma phenotypes (atopic vs. non-atopic), polytomous logistic regression analyses were done as described previously [49]. Based upon previous analyses in this population, *a priori* confounders for the association between helminth infections and outcomes were gender, age, maternal educational level, and parental asthma [17]. P values of <0.05 were considered statistically significant.

### Availability of supporting data

https://www.dropbox.com/s/vvbnbsqwcvr5nx1/Alcantara-Neves%20et%20al%20JACI%20%202012.pdf?dl=0

https://www.dropbox.com/s/x2bylt53la3gnqn/cav%20Inf%20immunity%202010.pdf?dl=0

https://www.dropbox.com/s/8ihrlmsjdomu0hh/Mendon%C3%A7a%20et%20al_PLOS%20TND_2012.pdf

https://dl.dropboxusercontent.com/u/11134490/Pontes-de-Carvalho_Frontiers%20immunology_2013.pdf.

## Abbreviations

CD 4^+^ CD25^+^: Clusters of differentiation expressed in Treg cells
FoxP3^+^: Transcription factor expressed by Treg cells
FcϵRI: High-affinity IgE receptor
IL-10: Interleukin 10
PBLs: Peripheral blood leukocytes
OR: Odds ratio
sIgE: Specific IgE against aeroallergens
SPT: Skin prick test
TGF-β: Transforming growth factor beta
Th1: T helper I lymphocytes
TH2: T helper 2 lymphocytes
Treg: Regulatory T lymphocytes.
